# Autoantibody repertoire in lupus nephritis: from pathogenic mechanisms to clinical integration for precision decision-making

**DOI:** 10.3389/fimmu.2026.1870304

**Published:** 2026-07-08

**Authors:** Ran Guo, Juan Xue, Rui Yu, Chiyuan Xue, Xiao Ma, Jin Liang, Baotong Yang, Hetao Chen, Tao Jiang, Chaojun Hu

**Affiliations:** 1 Luoyang key laboratory of transplantation and immunological studies for haematological diseases, Department of Clinical Laboratory, The First Affiliated Hospital, and College of Clinical Medicine of Henan University of Science and Technology, Luoyang, China; 2 Department of Rheumatology, Peking Union Medical College Hospital, Peking Union Medical College & Chinese Academy of Medical Sciences; Key Laboratory of Rheumatology & Clinical Immunology, Ministry of Education, Beijing, China

**Keywords:** autoantibody profile, biomarkers, lupus nephritis, pathogenesis, precision medicine

## Abstract

Lupus nephritis (LN) represents the most severe renal manifestation of systemic lupus erythematosus (SLE), and timely, risk-stratified intervention is essential to mitigate irreversible kidney damage and improve long-term clinical outcomes. The autoantibody repertoire not only constitutes a cornerstone of SLE diagnosis but also serves as a critical pathogenic link between systemic immune dysregulation and site-specific renal injury. This review synthesizes current evidence on established and emerging autoantibodies associated with LN, with an emphasis on their pathogenic contributions to three central mechanisms: immune complex deposition, complement system activation, and direct podocyte injury. We critically evaluate the clinical validity and utility of conventional serological markers, including anti-dsDNA and anti-C1q antibodies, for diagnosing LN, monitoring disease activity, and predicting renal flares or progression, while also elucidating the significance of distinct clinical phenotypes such as the antiphospholipid antibody profile. Furthermore, we discuss strategies for integrating multiple autoantibody indices to construct predictive models, as well as the application prospects of novel technologies enabling non-invasive liquid biopsy. Finally, the challenges and future directions of autoantibody-guided individualized therapy are discussed, with the goal of advancing precision clinical decision-making for LN.

## 1 Introduction

Lupus nephritis (LN) is the most common and severe renal complication of systemic lupus erythematosus (SLE) and remains the leading cause of progression to end-stage renal disease (ESRD) and death in these patients (1). Characterized by considerable clinical and histopathological heterogeneity, LN presents with a wide spectrum of manifestations—ranging from asymptomatic microscopic hematuria and microalbuminuria to overt nephrotic syndrome, acute kidney injury, and rapidly progressive glomerulonephritis (2, 3). Furthermore, LN often coexists with extrarenal lupus manifestations, including thrombotic microangiopathy (TMA), lupus myocarditis, and lupus encephalitis, and may overlap histologically or clinically with primary glomerulopathies such as IgA nephropathy, thereby complicating both diagnosis and therapeutic management (2, 4, 5). Therefore, elucidating the underlying pathogenic mechanisms and identifying robust, clinically actionable biomarkers are critical priorities for improving risk stratification and long-term outcomes in LN.

Autoantibody production is a central immunopathological hallmark of SLE, serving both as a diagnostic cornerstone and as a key effector mechanism driving renal injury. The pathogenesis of SLE involves interconnected processes, including loss of self-tolerance, immune system activation, and tissue inflammation. Within this framework, the innate immune system plays a pivotal role in inducing adaptive immune responses, thereby driving clonal expansion of autoreactive lymphocytes. This process leads to the generation of memory B cells and plasma cells, which secrete large quantities of autoantibodies targeting nuclear and cytoplasmic antigens. These autoantibodies form pathogenic ICs with cognate autoantigens; subsequent deposition in glomerular and tubulointerstitial compartments triggers renal damage through multiple interrelated pathways, including complement activation, Fcγ receptor−mediated inflammatory cell infiltration, and direct antibody−dependent cellular cytotoxicity (6). Although anti-dsDNA antibodies remain a canonical serological marker of LN, they exhibit polyspecific binding to diverse renal autoantigens, such as α-actinin, laminin, and collagen IV, and their precise pathogenic contributions continue to be investigated (7). Accumulating evidence indicates that, in addition to anti-dsDNA and anti-C1q antibodies, other autoantibody specificities, including anti−nucleosome antibodies (AnuA), anti−histone antibodies, antiphospholipid antibodies (aPLs), autoantibodies targeting complement components (e.g., anti−C3, anti−C4, anti−C1s, and anti−factor H), and antibodies against modified C−reactive protein (mCRP), contribute to renal inflammation and injury (8–11). Importantly, interindividual heterogeneity in autoantibody profiles correlates with distinct histopathological classes and clinical outcomes in LN, supporting their potential utility as stratification biomarkers for disease activity, treatment response, and long−term renal prognosis.

As our understanding of the pathogenesis of LN continues to evolve, the clinical utility of autoantibody profiling has expanded beyond its initial diagnostic role to encompass multiple critical domains: monitoring disease activity, predicting renal histopathological class, evaluating therapeutic response, and informing prognostic assessment. Consequently, systematic detection and integrative analysis of LN-associated autoantibody profiles can enhance diagnostic precision, support dynamic clinical decision-making, and facilitate the implementation of individualized, precision-based management strategies. In summary, this review systematically delineates the LN-associated autoantibody repertoire, explores its translation from pathogenic mechanisms to clinical decision-making, integrates current evidence, and proposes a framework for antibody-informed, risk-stratified patient management.

## 2 Clinical management challenges in lupus nephritis

LN is the most severe and life-threatening organ manifestation of SLE and remains the leading cause of ESRD and death in affected patients. Despite advances in immunosuppressive regimens, the global incidence of LN remains persistently high, and rates of treatment failure, CKD progression, and ESRD−related death are still unacceptably elevated, imposing substantial personal, clinical, and socioeconomic burdens (12). Notably, even with standardized first−line therapy, a considerable proportion of patients fail to achieve complete renal remission, and a significant subset experiences progressive eGFR decline, ultimately developing chronic kidney disease (CKD) or ESRD (13). European cohort studies further show that post−diagnosis renal function deterioration correlates strongly with patient−reported outcomes, including persistent pain, debilitating fatigue, reduced treatment satisfaction, and impaired physical functioning and health−related quality of life (14). Epidemiologically, LN exhibits marked disparities: African American, Hispanic, and Asian populations bear a disproportionately higher disease burden, characterized by earlier age of onset, increased prevalence, and poorer long−term renal survival (15). Moreover, patients with LN face multiple risks associated with CKD progression, including disabling or even fatal cardiovascular events, as well as secondary immunodeficiency and severe infections. These factors further compound disease complexity and clinical management difficulty (13).

The clinical management of LN faces several core challenges that limit long-term patient outcomes. First, early diagnosis remains difficult, as LN is clinically and histopathologically heterogeneous. According to the 2018 International Society of Nephrology/Renal Pathology Society(ISN/RPS)classification, LN comprises six pathological classes, with classes III and IV often exhibiting “full house” immunofluorescence and class V potentially coexisting with other classes (16). However, some patients present only with low-level proteinuria, lacking typical urinary sediment abnormalities or unexplained acute kidney injury; notably, even among SLE patients with proteinuria below the guideline-recommended biopsy threshold (<1000 mg/24h), renal biopsy reveals histological evidence of LN in up to 76% of cases, including severe proliferative or membranous lesions, underscoring the inadequacy of relying solely on traditional clinical indicators for early diagnosis (17). Second, disease activity assessment is imprecise, as current reliance on proteinuria, serum creatinine, complement levels, and anti-dsDNA antibodies correlates imperfectly with renal histological activity, limiting the ability to reflect true intrarenal inflammation (12). For example, in active LN patients, the burden of type 2 SLE symptoms (e.g., fatigue, fibromyalgia) is similar to that in non-nephritis patients, whereas type 1 (classic inflammatory) disease activity differs, suggesting discordance between symptomatic and renal inflammatory activity and increasing clinical complexity (18). Third, treatment response varies substantially among individuals; although classic induction regimens (mycophenolate mofetil, low-dose cyclophosphamide) and newer targeted agents (belimumab, voclosporin) have been introduced, complete remission rates remain modest (40–60%), and relapse rates are persistently high (19). Patients exhibit marked heterogeneity in response to the same regimen, with some showing poor or no response to standard therapy, leading to ongoing disease activity or rapid progression. Fourth, relapse prediction is inadequate, as LN is highly prone to recurrence yet no reliable biomarkers exist to identify patients at high risk of relapse after remission, precluding targeted preventive interventions (12). Collectively, these clinical management challenges contribute to uncertainty in long-term LN outcomes, underscoring the urgent need for more precise diagnostic tools and individualized treatment strategies. Autoantibodies serve as key molecular mediators linking systemic immune dysregulation to target-organ injury; therefore, a mechanistic understanding of their pathogenic roles is central to overcoming the challenges outlined above.

## 3 The multi-pathway injury mechanism of lupus nephritis driven by autoantibodies

### 3.1 Immune complex-mediated injury: deposition, activation and inflammatory amplification

The core mechanism of renal injury in LN lies in the deposition and cascade activation of immune complexes (ICs) within the kidney, primarily achieved through two pathways: deposition of circulating ICs and *in situ* IC formation. Circulating ICs, predominantly composed of anti-dsDNA antibodies bound to nuclear antigens (e.g., histones, nucleosomes), represent the canonical pathogenic route (20). These complexes preferentially deposit in the glomerular mesangial and subendothelial compartments upon renal filtration, triggering local leukocyte infiltration, cytokine release, and endothelial activation (21). Renal biopsy studies consistently demonstrate widespread IC deposition, not only within glomeruli but also extending into tubulointerstitial and vascular compartments—defining the hallmark “full-house” immunofluorescence pattern (co−deposition of IgG, IgA, IgM, C1q, and C3) that underpins LN diagnosis and classification; Notably, this pattern remains diagnostically decisive even in seronegative patients (i.e., those lacking detectable serum anti-dsDNA or hypocomplementemia), reinforcing its status as a tissue−level gold standard (22, 23).

Concurrently, *in situ* IC formation occurs when circulating autoantibodies bind directly to intrinsic glomerular antigens, or to antigens aberrantly exposed or deposited (“implanted”) on the glomerular basement membrane or podocyte surface, leading to localized IC deposition (24). Identified target antigens include laminin, α−actinin, and phosphatidylserine. Of note, anti−phosphatidylserine IgG ICs not only deposit within glomeruli but also impair macrophage phagocytic function by inducing oxidative stress, thereby amplifying renal inflammation (25). Similarly, autoantibodies targeting monomeric C−reactive protein (mCRP) mediate *in situ* IC formation in the kidney; their serum levels correlate significantly with clinical disease activity, severity of tubulointerstitial injury, therapeutic response, and long−term renal prognosis (10). These two pathogenic mechanisms frequently operate synergistically, culminating in substantial intraglomerular IC accumulation.

IC deposition is not a terminal event but rather the initiating trigger for complement cascade activation. Early complement components facilitate clearance of ICs and cellular debris, exerting a protective homeostatic function; however, dysregulated complement activation, particularly hyperactivation of the alternative pathway, drives renal inflammation and tissue injury (26). Complement activation generates large amounts of chemokines and anaphylatoxins, including C5a, which recruit neutrophils and monocytes/macrophages into the renal parenchyma and stimulate intrinsic renal cells to secrete pro−inflammatory mediators, thereby establishing a self−amplifying inflammatory cascades (27). Moreover, IC engagement of Fcγ receptors (FcγRs) on macrophages induces metabolic reprogramming toward aerobic glycolysis, a process orchestrated by mTOR and HIF−1α signaling pathways, and this metabolic shift is essential for the production of key pro−inflammatory cytokines such as IL−1β (28). Infiltrating immune cells engage in dynamic, bidirectional crosstalk with resident renal cells, including podocytes, mesangial cells, and tubular epithelial cells. Notably, podocytes actively contribute to local immune dysregulation by upregulating MHC class II molecules and co−stimulatory ligands (e.g., CD80/CD86), enabling antigen presentation and T−cell co−stimulation, which perpetuates autoimmune injury (29). Similarly, tubulointerstitial inflammation is sustained by organized lymphoid−like interactions among T follicular helper (Tfh) cells, B cells, and dendritic cells within the kidney (30). Collectively, IC deposition and downstream effector activation constitute an integrated pathogenic axis, spanning autoantibody−driven IC formation, complement initiation, leukocyte recruitment, metabolic reprogramming of innate immune cells, and adaptive immune cell crosstalk, that serves as the central orchestrator of renal injury in LN.

### 3.2 The multiple roles of the complement system: activation cascades, tissue damage, and clinical surveillance

In LN, dysregulated complement activation is a central pathogenic driver, primarily triggered by glomerular IC deposition via the classical pathway: C1q binds to IgG Fc regions, initiating the proteolytic cascade that generates the C3 convertase (31). Critically, anti−C1q autoantibodies amplify this activation (32). Beyond the classical pathway, robust evidence now implicates both the alternative and lectin pathways in LN pathogenesis. Elevated renal expression of factor B and factor D, key components of the alternative pathway, correlates with histologic activity and predicts poor renal outcomes (33). Similarly, mannose−binding lectin (MBL) and ficolins activate the lectin pathway in LN kidneys, although their net effect remains context−dependent: potentially protective during early immune surveillance but damaging upon sustained activation in inflamed tissue (31). This convergent, multi−pathway activation establishes a self−reinforcing complement pathological network. Most significantly, intrarenal complement synthesis is not merely a passive reflection of systemic inflammation but an active, disease−specific contributor. Transcriptomic and spatial proteomic analyses demonstrate upregulated expression of C1q, C3, factor B, properdin, and MBL specifically within the glomerular and tubulointerstitial compartments of LN biopsies, levels that strongly correlate with eGFR decline, interstitial fibrosis scores, and resistance to standard immunosuppression (34). Collectively, this multilayered, interdependent activation of complement pathways forms the core complement−mediated pathological network in LN.

Upon complement cascade activation, a series of biologically potent effectors is generated, most notably the anaphylatoxins C3a and C5a, and the terminal membrane attack complex (C5b-9), each mediating distinct yet synergistic mechanisms of renal injury. First, C5a acts as a master chemoattractant, recruiting neutrophils, monocytes, and macrophages into glomerular and peritubular capillaries and initiating a localized inflammatory infiltrate (27). These recruited leukocytes release reactive oxygen species (ROS), neutrophil elastase, and matrix metalloproteinases (MMPs), directly degrading the glomerular basement membrane, disrupting slit diaphragm integrity, and inducing podocyte apoptosis. Second, C5b-9 inserts into the plasma membranes of intrinsic renal cells, including podocytes, endothelial cells, and tubular epithelial cells, leading to sublytic calcium influx, mitochondrial dysfunction, and pro−fibrotic signaling (35). Third, a pathogenic feedback loop exists between neutrophil extracellular traps (NETs) and complement: NET components (e.g., histones, LL−37) activate the classical and alternative pathways, while C5a potently stimulates NETosis, thereby creating a self−amplifying cycle that sustains inflammation, exposes nuclear autoantigens, and promotes IC formation (36). Furthermore, C3a and C5a exert direct pro−inflammatory signaling via their cognate G−protein−coupled receptors (C3aR and C5aR) on renal parenchymal and immune cells. Notably, intrarenal C5aR expression—markedly elevated in classes III and IV LN—strongly correlates with delayed eGFR recovery after induction therapy and predicts progression to CKD, establishing the C5a–C5aR axis as a key determinant of treatment response and long−term renal outcomes (37).

Clinically, complement system hyperactivation is most directly reflected by depletion of serum complement components, with declining C3 and C4 levels established as classic serological markers for evaluating LN disease activity. Longitudinal declines in serum C3 and C4 correlate strongly with histologic flare, rising proteinuria, and declining eGFR, whereas their normalization during therapy reliably predicts sustained remission and reduced relapse risk (27, 38); thus, serial measurement of C3 and C4 remains a routine clinical tool for monitoring treatment response and guiding immunosuppressive adjustments. However, total C3/C4 quantification reflects net balance of hepatic synthesis and peripheral consumption, rendering it relatively insensitive to early or compartmentalized activation. Complement activation fragments offer superior specificity and dynamic range: plasma levels of C3dg, iC3b, and C4d rise significantly in active LN, and their ratios to intact C3 (e.g., C3dg/C3) or C4 (e.g., C4d/C4) provide real−time readouts of classical and alternative pathway flux (39, 40), C4d deposition has been validated as a tissue−based biomarker in SLE/LN, supporting diagnostic classification and disease monitoring (41). Circulating anti−C3b autoantibodies are detectable in approximately 30% of LN patients and correlate with histopathological severity, renal flare risk, and treatment resistance (42). Moreover, cell−bound complement fragments, including erythrocyte−bound C4d (EC4d), platelet−bound C4d (PC4d), and B−cell-bound C4d (BC4d), are effective biomarkers, with EC4d showing stronger correlation with lupus disease activity than low plasma complement levels (40). Critically, urinary complement activation products (C3a, C5a, Ba, and soluble C5b−9) are markedly elevated in active LN and correlate with histopathologic features of thrombotic microangiopathy, endothelial injury, and tubulointerstitial inflammation, thereby enabling non−invasive, kidney−specific assessment of intrarenal complement activation (43).

Collectively, the complement system plays multifaceted pathogenic roles in lupus nephritis, initiating immune injury, amplifying inflammatory cascades, and guiding clinical decision-making through biomarker-based monitoring. Accordingly, complement-derived biomarkers are shifting LN surveillance away from conventional functional parameters (e.g., serum creatinine, proteinuria) toward mechanism−informed, tissue− and pathway−specific indicators, thereby enabling more precise risk stratification and targeted therapeutic interventions.

### 3.3 Direct assault on kidney intrinsic cells: from passive targets to active players

The classical paradigm attributes LN pathogenesis primarily to IC deposition within the glomerular basement membrane, followed by complement activation and downstream inflammatory cascades (44). However, emerging evidence supports an IC−independent pathway involving direct autoantibody−mediated injury to intrinsic renal cells, including podocytes, mesangial cells, and tubular epithelial cells, thereby offering a mechanistic explanation for the clinical and histopathological heterogeneity of LN (45). One study identified that the peptide HU1, derived from a conserved bacterial biofilm component, induces a novel autoantibody that not only correlates positively with LN incidence but also cross−reacts with protein disulfide isomerase (PDI), a cell−surface enzyme on renal cells. By inhibiting PDI enzymatic activity, this autoantibody directly impairs cellular homeostasis (46). Such direct targeting appears especially relevant in membranous LN, where anti−podocyte antibodies may induce cytoskeletal disorganization, apoptosis, or functional impairment of podocytes, culminating in severe proteinuria. The therapeutic efficacy of the novel calcineurin inhibitor voclosporin, which acts predominantly by stabilizing podocyte architecture and function, further underscores the pivotal role of podocytes as primary targets in this direct injury axis (47).

This direct cytotoxic mechanism explains atypical LN presentations, such as marked proteinuria without prominent IC deposition on renal biopsy (48). In such cases, autoantibodies targeting cell-surface antigens on intrinsic renal cells likely serve as primary pathogenic drivers. Importantly, intrinsic renal cells are not merely passive targets but actively participate in disease pathogenesis through dynamic molecular regulation. Under inflammatory stimulation, mesangial cells upregulate the long non−coding RNA NEAT1, which modulates the miR−146b/TRAF6/NF−κB axis to amplify autocrine inflammation and release pro−inflammatory mediators (49). Similarly, the circular RNA circRTN4 is transferred from monocytes to mesangial cells via exosomes, where it suppresses miR−513a−5p and activates the FN−dependent pathway, promoting cellular proliferation and pathological extracellular matrix accumulation (50). Upon IL−22 stimulation, renal tubular epithelial cells activate the STAT3 signaling cascade, increasing transcription and secretion of the chemokines CCL2 and CXCL10, which recruit macrophages into the renal interstitium (51). Collectively, autoantibody−mediated direct cytotoxicity and cell−intrinsic dysregulated signaling networks synergistically drive renal lesion progression.

Moreover, genetic determinants within renal cells govern their susceptibility to immune−mediated damage. VANGL1 has been identified as a renal−cell−autonomous genetic risk factor for IC deposition in LN, suggesting that the genetic background of intrinsic renal cells critically influences their vulnerability to autoantibody attack (48). Additionally, ferroptosis—an iron−dependent, lipid peroxidation−driven form of regulated cell death—has been implicated in tubular epithelial injury in LN, revealing a previously underappreciated cell−autonomous death pathway contributing to renal parenchymal damage (52). In summary, direct autoantibody−mediated cytotoxicity against intrinsic renal cells and the consequent dysregulation of intracellular signaling pathways constitute a key pathogenic axis in LN. Elucidating this mechanism not only advances our understanding of LN heterogeneity but also provides a rational foundation for targeted therapeutics aimed at preserving renal cell integrity and selectively inhibiting pathogenic signaling cascades. For example, pharmacological inhibition of NF−κB−inducing kinase (NIK) or blockade of CD11b−mediated leukocyte adhesion and activation represents a promising frontier in precision nephrology, shifting therapeutic paradigms from broad−spectrum immunosuppression toward mechanism−informed, cell−type−specific interventions (53, 54).

**Figure 1:**
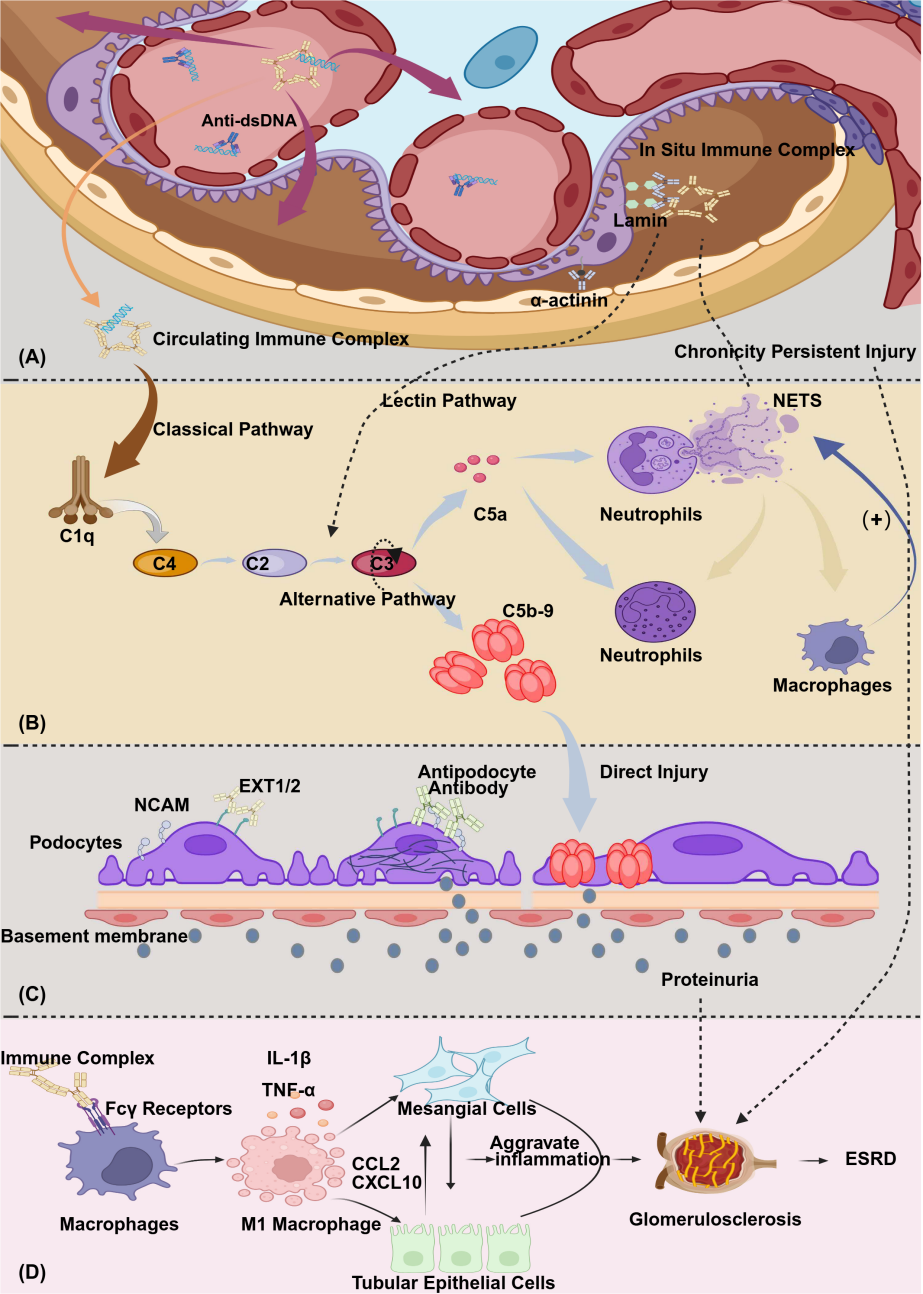
Autoantibody−driven multi−pathway injury mechanisms in lupus nephritis. **(A)** Immune complex deposition: Circulating immune complexes deposit in the subendothelial/mesangial regions of glomeruli; autoantibodies bind to podocyte surface antigens to form *in situ* immune complexes. **(B)** Complement activation and inflammatory amplification: Immune complexes activate the classical complement pathway, generating C5a and the C5b−9 membrane attack complex; NETs form a positive feedback loop that amplifies complement activation. **(C)** Direct podocyte injury: Anti−podocyte antibodies directly injure podocytes, leading to cytoskeletal disruption, foot process effacement, and proteinuria. **(D)** Inflammatory amplification and outcome: Immune complexes activate macrophages (M1 polarization) and resident renal cells via Fcγ receptors, releasing inflammatory cytokines, ultimately resulting in glomerulosclerosis, interstitial fibrosis, and end−stage renal disease.

In summary, LN renal injury results from three intertwined pathways: immune complex deposition, complement cascade amplification, and direct autoantibody attack on resident renal cells (**Figure 1**). Understanding these connections provides the pathophysiological basis for using autoantibodies in LN diagnosis, classification, and treatment.

## 4 Clinical utility of the autoantibody repertoire in lupus nephritis

Autoantibodies are integral to the 2019 ACR/EULAR classification criteria for SLE and serve as foundational diagnostic biomarkers (55). In lupus nephritis, the most severe organ manifestation, specific autoantibody features, including isotype, titer, antigenic specificity, IgG subclass distribution, and glycosylation status, correlate strongly with pathogenic mechanisms, histopathological class, disease severity, and renal outcomes (**Table 1**). Extended autoantibody profiling has identified clinically distinct SLE endotypes; notably, Ro52−specific autoantibodies are associated with increased risk of renal involvement (56, 57). In pediatric and adolescent−onset LN, concurrent anti−dsDNA and anti−Ro/SSA positivity independently predicts accelerated progression to ESRD (56). Thus, comprehensive analysis of the autoantibody repertoire provides mechanistic insight into interpatient heterogeneity in renal injury. Contemporary practice is moving beyond binary serological assessment (“present/absent”) toward dynamic, quantitative repertoire monitoring, recognizing that antibody isotype, target specificity, quantity, titer, IgG subclass, and glycosylation patterns together constitute the “autoantibody repertoire” that shapes disease course (58). Integrating autoantibody repertoire data into LN clinical management supports earlier diagnosis, biologically informed risk stratification, and mechanism−targeted therapeutic decisions.

**Table 1:** Clinical utility of autoantibodies in lupus nephritis.

Autoantibody	Target antigen	Clinical value	Pathogenic mechanism	Recommended/current detection methods
Anti-dsDNA	dsDNA	Disease activity monitoring, relapse prediction, high-risk identification	Forms immune complexes depositing on glomerular basement membrane, activates classical complement pathway; cross-reacts with intrinsic glomerular antigens	Farr assay (gold standard), Crithidia luciliae IFA, ELISA, CLIA
Anti-C1q	C1q	Activity assessment, relapse prediction, distinguishing proliferative from non-proliferative LN	Binds with high affinity to glomerular-deposited C1q, enhances classical pathway activation, exacerbates complement consumption and inflammation	ELISA (recombinant or native C1q antigen)
Anti-Sm	Sm protein	Marker of disease severity, indicator of aggressive renal involvement	No direct renal target but associated with severe systemic activity; indirectly contributes via systemic immune dysregulation	ELISA, line immunoassay (LIA)
Anti-RNP	U1-RNP	Pathological subtype hint, type I interferon pathway association, mixed connective tissue disease phenotype	Associated with enhanced type I interferon signaling; commonly seen in overlapping MCTD features	ELISA, LIA
Anti-nucleosome (AnuA)	Nucleosome	Independent predictor of renal injury	Binds to heparan sulfate proteoglycans on glomerular basement membrane via histone cationic domains, leading to immune complex deposition	ELISA (histone-DNA complex)
Antiphospholipid antibodies (aPLs)	Phospholipids/β2GPI (LA, aCL, anti-β2GPI)	Early warning of TMA, poor prognosis, guidance for anticoagulation therapy	Activates vascular endothelial cells, induces prothrombotic and proinflammatory phenotype via the alternative complement pathway, leading to microvascular thrombosis	LA: dilute Russell viper venom time (dRVVT)/sensitive PTT; aCL/anti-β2GPI: ELISA, CLIA, ALBIA
Anti-podocyte antibodies	EXT1/2, NCAM1 (class V LN)	Precision subtyping, guidance for B cell-targeted therapy	Forms *in situ* immune complexes, directly injures podocytes	Research use: IFA or ELISA with recombinant antigens
Anti-C1s	C1s serine protease	Enhances diagnostic specificity in multi-antibody panels	Amplifies classical complement pathway activation; synergistic with anti-dsDNA and anti-C1q	Research use: ELISA (N-terminal domain specific)
Anti-α-enolase	α-enolase	Emerging biomarker	Recognizes modified epitopes (citrullination, oxidation), participates in NETosis-associated inflammation	Research use: ELISA
Anti-phosphatidylserine (anti-PS)	Phosphatidylserine	Amplifies inflammation, worsens renal injury	Forms immune complexes depositing in glomeruli; induces oxidative stress and impairs macrophage phagocytosis	Research use: ELISA

IFA, immunofluorescence assay; CLIA, chemiluminescence immunoassay; ALBIA, addressable laser bead immunoassay; dRVVT, dilute Russell viper venom time; PTT, partial thromboplastin time;”Research use” indicates that the assay is not yet widely standardized or clinically validated for routine use; these markers are primarily investigational.

### 4.1 Monitoring disease activity: anti-dsDNA and anti-C1q autoantibodies

Anti-dsDNA antibodies exhibit high specificity for SLE, and their seropositivity is robustly associated with an elevated risk of LN. Clinical cohort studies consistently demonstrate that anti-dsDNA−positive patients have significantly higher SLEDAI scores and a substantially increased incidence of renal involvement. Consequently, anti-dsDNA serostatus has been established as a cornerstone serological biomarker for stratifying LN risk in clinical practice (20). Nevertheless, rare but clinically important cases of biopsy−confirmed LN occur in the absence of conventional serological markers, including anti-dsDNA negativity and normocomplementemia, underscoring the limitations of serology alone in disease classification and reinforcing the indispensable role of renal biopsy in diagnostically ambiguous or seronegative presentations (59).Changes in anti-dsDNA antibody titers correlate strongly with renal disease activity, particularly in proliferative LN (ISN/RPS classes III and IV), and rising titers frequently herald renal flare or histological recurrence. As such, longitudinal anti-dsDNA monitoring constitutes a cornerstone serological tool for assessing disease activity in LN (60, 61). Evidence further indicates that elevated anti-dsDNA titers and concomitant C3 hypocomplementemia at the time of kidney transplantation are independent serological risk factors for early post−transplant LN recurrence (62).

As described in Section 3.1, anti-dsDNA antibodies form circulating immune complexes with cell−free DNA, activate complement, and bind to NETs (63, 64). oreover, acquired deficiency ofdeoxyribonuclease 1−like 3 (DNASE1L3) results in impaired clearance of cell−free DNA, fostering high−affinity anti−dsDNA autoantibodies correlated with disease severity (65).

Anti-C1q antibodies act as potent amplifiers of complement-mediated injury in LN. Their primary pathogenic mechanism involves high-affinity binding to C1q molecules already deposited within glomerular immune complexes, thereby potentiating classical pathway activation, exacerbating local inflammation, and promoting further immune complex deposition in the glomerular basement membrane. Serum anti−C1q levels correlate inversely with complement C3 and C4, suggesting their contribution to systemic and intrarenal complement consumption (61). At the molecular level, antibodies targeting specific C1q epitopes, such as A08, can interfere with normal C1q function and thereby affect classical pathway regulation (66). Notably, in pediatric LN, elevated anti−C1q titers correlate significantly with histopathological features of microvascular thrombosis and higher renal activity index scores, suggesting a synergistic pathogenic role with anti−β2−glycoprotein I (anti−β2GPI) antibodies in promoting intraglomerular thromboinflammation (67).

Clinical evidence supports a strong association between anti−C1q antibodies and active proliferative LN (ISN/RPS classes III and IV), with consistently high sensitivity and specificity (68). In distinguishing active LN from SLE patients without renal involvement, anti−C1q seropositivity demonstrates diagnostic performance comparable to and in some cohorts superior to that of anti−dsDNA antibodies (69). Among pediatric patients, anti−C1q testing achieves a specificity of 85% for identifying active LN (70). Serum anti−C1q titers correlate significantly not only with the SLEDAI but also with histopathological activity scores, including the renal activity index, reflecting intrarenal inflammatory burden (71). Multivariate regression analyses confirm anti−C1q serostatus as an independent predictor for differentiating proliferative from non−proliferative LN subtypes. Moreover, elevated baseline anti−C1q levels were identified as a superior independent predictor of renal flares (OR 9.07; P = 0.0057) compared to traditional markers (72). From a clinical management perspective, serial anti−C1q quantification closely tracks LN disease activity: post−treatment declines in titer strongly parallel achievement of clinical remission, whereas persistently elevated levels (e.g., >40 U/mL) constitute an independent risk factor for renal flare or histological recurrence (72, 73). Sustained seronegativity or persistent low−level titers following therapy are associated with prolonged renal remission and improved long−term renal outcomes. A separate large prospective cohort confirmed that baseline anti−C1q, together with anti−dsDNA, can noninvasively predict proliferative LN; in patients with this diagnosis, higher baseline anti−C1q predicted complete response (AUC 0.72) better than baseline proteinuria (AUC 0.59) (74). Research indicates that anti−C1q antibody is the optimal biomarker for LN activity, with a negative predictive value of 92%; its negativity renders active nephritis highly unlikely (75). In conclusion, incorporating anti−dsDNA and anti−C1q antibodies into routine clinical monitoring enables precise risk stratification, early detection of disease recurrence, and objective assessment of treatment response in LN.

### 4.2 Indicators of disease severity: anti-Sm, anti-RNP, and anti-nucleosome antibodies

Anti-Sm and anti-U1-RNP antibodies are closely associated with multi-system severe involvement and specific clinical phenotypes. In juvenile-onset SLE (JSLE), anti-Sm positivity significantly correlates with serositis, cutaneous and mucocutaneous lesions, constitutional symptoms, and neuropsychiatric manifestations; anti-U1-RNP positivity is significantly linked to oral ulcers, Raynaud’s phenomenon, hematologic abnormalities, neuropsychiatric involvement, and thromboembolic events. Both autoantibodies correlate with moderate-to-high SLEDAI scores and independently predict elevated disease activity (76). Among pregnant individuals with SLE, the presence of either antibody is associated with adverse pregnancy outcomes, including small-for-gestational-age (SGA) neonates, and both constitute independent predictors of SGA (77, 78). A retrospective cohort study from Saudi Arabia reported that concurrent positivity for anti-Sm, anti-Ro, and anti-U1-RNP antibodies conferred a significantly elevated risk of incident proteinuria within five years of SLE diagnosis, suggesting that this triad may identify a subgroup at accelerated risk for LN development (79). Furthermore, in patients with SLE-associated pulmonary arterial hypertension (SLE-PAH), the anti-Sm/anti-U1-RNP-positive immunoserological cluster (designated CL1) exhibits more pronounced baseline hemodynamic impairment, characterized by significantly higher mean right ventricular systolic pressure, and a greater likelihood of requiring dual-targeted PAH therapy compared with other antibody-defined clusters (80).

Although anti-Sm autoantibodies lack direct renal targets, their seropositivity is consistently associated with more severe and treatment−refractory lupus nephritis. In large multicenter pediatric SLE cohorts, anti−RNP autoantibodies show no significant association with early−onset LN (81); however, anti−U1−RNP positivity frequently coincides with clinical features overlapping with mixed connective tissue disease (MCTD). In such cases, renal involvement typically presents as membranous nephropathy, characterized by subepithelial immune deposits, and is mechanistically linked to heightened type I interferon signaling activity (82).

Anti-nucleosome autoantibodies (AnuA) recognize nucleosomes, DNA−histone complexes released during apoptotic cell death, which serve as fundamental autoantigens in SLE. In LN, nucleosome–AnuA ICs bind electrostatically to negatively charged heparan sulfate proteoglycans in the glomerular basement membrane via histone−derived cationic domains; this deposition subsequently recruits and activates the classical complement pathway, leading to immune−mediated glomerular injury (83). Thus, AnuA detection is not only a highly specific serological marker for SLE diagnosis but also an early indicator of incipient renal immune dysregulation, offering mechanistic insight into the transition from systemic autoimmunity to organ−specific inflammation (84). Clinically, AnuA demonstrates robust discriminative capacity in distinguishing active LN from non−renal SLE. Elevated AnuA titers correlate significantly with heightened systemic inflammatory burden (reflected by higher SLEDAI scores, hypocomplementemia, and an increased interferon signature) and with greater histopathological activity on renal biopsy (85, 86). Large−scale prospective cohort studies further establish AnuA as an independent predictor of incident renal damage (hazard ratio (HR) = 2.51), with synergistic risk amplification observed in patients co−positive for anti−dsDNA antibodies (HR = 3.19) (87).

Although anti-Sm, anti-RNP, and anti-nucleosome autoantibodies target distinct nuclear antigens, their combined assessment provides complementary prognostic information for evaluating LN severity and predicting renal outcomes. Multiplex serological profiling integrating these markers enhances risk stratification beyond single−analyte approaches.

### 4.3 Antiphospholipid antibody profile: early warning for special phenotypes

The antiphospholipid antibody (aPL) profile, including lupus anticoagulant (LA), IgG/IgM anticardiolipin antibodies (aCL), and IgG/IgM anti−β2−glycoprotein I antibodies (anti−β2GPI), is a well−validated serological predictor of thrombotic microangiopathy (TMA) in LN. Among patients with biopsy−confirmed renal TMA, the overall prevalence of aPL positivity is approximately 24.4%, with LA showing the highest detection rate (18.9%), followed by aCL and anti−β2GPI (each ~4.0%) (88). Renal biopsies of aPL−positive LN patients commonly reveal acute or chronic TMA features, including glomerular intracapillary thrombosis and endothelial injury, which may occur without concurrent typical proliferative lesions (89, 90). Clinically, LN patients with aPL−associated TMA exhibit a more aggressive disease course, reduced response to conventional immunosuppression, and significantly worse renal outcomes—including lower rates of renal function recovery and higher cumulative incidence of ESRD. A retrospective analysis showed that among patients with ISN/RPS class IV LN, those with TMA had a markedly higher proportion of dialysis dependence during follow−up (37.5% vs. 0%) (4). Robust evidence confirms that renal TMA is an independent predictor of impaired renal recovery in LN, strongly associated with accelerated eGFR decline and reduced renal survival (91).

This adverse prognosis reflects a distinct pathogenic mechanism: anti−β2GPI antibodies bind to β2GPI exposed on activated endothelial cells, inducing a prothrombotic and proinflammatory endothelial phenotype. This triggers complement activation, particularly via the alternative pathway, as well as platelet adhesion and microvascular fibrin deposition, culminating in widespread microthrombosis (92, 93). In catastrophic antiphospholipid syndrome (CAPS), this process may become systemic, precipitating multiorgan failure (94). Consequently, immunosuppression alone is insufficient to halt thromboinflammatory injury, and integrated strategies targeting both autoimmunity and coagulation dysregulation are warranted. A recent meta−analysis demonstrates that aPL positivity confers a three− to five−fold increased risk of acute and chronic renal microvascular injury, with LA and IgG aCL showing the strongest independent associations (95). For aPL−positive LN patients, especially those with a high−risk profile (e.g., LA positivity, triple positivity, or anti−β2GPI positivity), anticoagulation should be added to immunosuppressive therapy (96, 97). Evidence suggests that vitamin K antagonists (e.g., warfarin) may offer superior antithrombotic efficacy over direct oral anticoagulants (DOACs) in this population, particularly in the setting of high−titer LA or triple positivity (98). Furthermore, complement−targeted therapies, such as the anti−C5 monoclonal antibody eculizumab, have shown clinical benefit in refractory aPL−associated TMA by inhibiting complement−mediated endothelial damage and microthrombosis (99, 100). Thus, systematic integration of aPL testing into the LN diagnostic and prognostic workflow enables earlier identification of patients at high risk for thrombotic renal injury, supports timely, mechanism−informed therapeutic intervention, and ultimately contributes to improved long−term renal survival.

### 4.4 Emerging autoantibody profiles: expanding mechanisms and translational frontiers

#### 4.4.1 Anti−podocyte antibodies: new mechanisms for precision subtyping in MLN

Recent advances have established autoantibodies targeting podocyte-specific surface antigens as central mediators of membranous lupus nephritis (ISN/RPS class V LN). In primary membranous nephropathy (MN), circulating autoantibodies against constitutively expressed podocyte membrane antigens, including M−type phospholipase A2 receptor (PLA2R1) and thrombospondin type−1 domain−containing 7A (THSD7A), induce *in situ* immune complex formation, complement activation (predominantly via the lectin and alternative pathways), and downstream podocyte cytoskeletal disruption, culminating in selective proteinuria (101, 102). Analogously, autoantibodies targeting exostosin−1 and −2 (EXT1/EXT2) and neural cell adhesion molecule 1 (NCAM1) have been identified in subsets of patients with class V LN (103). These findings provide robust evidence that antigen−specific autoimmunity, rather than nonspecific immune complex trapping, drives subepithelial immune deposit formation and glomerular basement membrane injury in membranous LN, extending the “autoantibody−mediated podocytopathy” framework from primary MN to SLE−related glomerular disease.

In transgenic mice expressing human PLA2R1, spontaneous generation of anti−human PLA2R1 antibodies leads to histologically and clinically authentic membranous nephropathy, characterized by nephrotic−range proteinuria, granular IgG deposits along the glomerular basement membrane, and subepithelial electron−dense deposits (104). By contrast, Rag2−/− mice, which lack mature B and T lymphocytes, fail to produce anti−PLA2R1 antibodies and remain phenotypically normal, providing definitive genetic evidence for antibody−dependent pathogenesis. *In vitro*, affinity−purified anti−PLA2R1 IgG from patients with idiopathic MN directly induces podocyte injury, evidenced by downregulation of podocin, actin cytoskeletal disorganization, and caspase−1−dependent pyroptosis (105). Similarly, IgG fractions from LN patients induce podocyte pyroptosis and functional impairment, confirming cross−disease pathogenic relevance (106). Notably, in *de novo* MN following kidney transplantation, podocyte−expressed PLA2R1 is consistently detected in biopsy specimens and frequently co−occurs with features of antibody−mediated rejection, demonstrating that antigen−specific autoimmunity can persist and drive injury despite maintenance immunosuppression (107). Collectively, these data establish that anti−podocyte autoantibodies, whether arising in primary or SLE−associated membranous nephropathy, mediate podocyte dysfunction and severe proteinuria through convergent mechanisms: direct antigen binding and disruption of critical podocyte proteins, cytoskeletal disorganization, induction of pyroptosis, and complement activation.

Although detection of anti−podocyte antibodies has become an important diagnostic and monitoring tool in primary MN, its application in LN remains largely confined to research settings (101, 108). In the future, detection of specific anti−podocyte antibodies (e.g., anti−EXT1/2 or anti−NCAM) in patients with class V LN may become a key biomarker for distinguishing classic immune complex−driven LN from podocyte−targeting antibody−driven subtypes (103). This precise classification is crucial for clinical decision−making: patients with high antibody titers may benefit from B−cell or plasma cell−directed therapies, such as rituximab, or from combined therapeutic plasma exchange to rapidly clear pathogenic antibodies (109, 110). Therefore, in−depth exploration of the anti−podocyte antibody profile in class V LN not only deepens our understanding of disease heterogeneity but also promises to translate mechanistic insights into precise clinical decision−making, enabling biomarker−based subtyping, prognosis assessment, and individualized targeted therapy.

#### 4.4.2 Other novel autoantibody targets

In addition to the classic anti-dsDNA antibodies, novel autoantibodies targeting specific renal structural components, complement system proteins, and vascular endothelium are emerging as the frontier of translational research in LN. Autoantibodies against α-enolase have been detected in LN patients, with the IgG2 subtype being particularly prominent. Under inflammatory conditions, α-enolase undergoes post-translational modifications such as citrullination, oxidation, and phosphorylation, which may serve as new epitopes recognized by autoantibodies, suggesting its role in specific renal injury mechanisms (111). Additionally, autoantibodies against phosphatidylserine (PS) have been confirmed to be produced by B1a cells and activated through the Toll-like receptor (TLR)/Syk signaling pathway, directly participating in the pathogenesis of LN. In lupus model mice and LN patients, the serum levels of PS-specific IgG increase, and they accumulate in renal biopsy tissues. Transferring PS-specific IgG from SLE patients to recipient mice can trigger lupus-like glomerular immune complex deposition (112).

Beyond the well-characterized anti-C1q autoantibodies, anti-C1s protease autoantibodies, predominantly targeting the N-terminal domain of the C1s serine protease, are significantly elevated in patients with active lupus nephritis (LN), with serum levels correlating strongly with histologic activity indices and renal SLEDAI scores (9). When incorporated into a multimarker panel alongside anti-double-stranded DNA (anti-dsDNA) and anti-C1q antibodies, anti-C1s detection achieves 94.6% specificity and 80% positive predictive value for identifying active proliferative LN (ISN/RPS Classes III and IV), thereby enhancing diagnostic precision beyond single-marker approaches. In the MRL/lpr murine lupus model, properdin deficiency markedly attenuates disease severity: properdin−/− mice exhibit significantly lower anti-dsDNA antibody titers, reduced glomerulonephritis activity scores, and preserved renal function (lower serum creatinine and BUN) compared with wild-type controls (113). In contrast, anti−complement factor H antibodies are associated with milder renal injury and may exert a protective role (11, 114). Anti−Tyro3 receptor autoantibodies are significantly elevated in the serum of treatment−naïve patients with newly diagnosed SLE and correlate positively with SLEDAI scores; they also inhibit macrophage efferocytosis (115).

A retrospective cohort study investigated the association between serum autoantibody profiles and soluble vascular cell adhesion molecule-1 (sVCAM-1), a validated biomarker of endothelial activation, in patients with SLE. Elevated anti-Sm antibody titers were significantly associated with impaired endothelial function, as reflected by higher sVCAM-1 concentrations and reduced flow-mediated dilation; conversely, elevated anti-dsDNA antibody levels correlated inversely with sVCAM-1 (116).

Autoantibodies against intrarenal antigens, complement regulators, and endothelial receptors may delineate distinct immunopathological subtypes of LN and could be developed into novel diagnostic and therapeutic panels for precision medicine. In addition to antibody isotype and titer, IgG subclass distribution and glycosylation modifications further refine the resolution of the autoantibody repertoire. Elevated IgG1 anti-dsDNA levels independently predict renal flare (91% vs. 45%; P = 0.02), whereas IgG2 anti-nucleosome antibodies demonstrate superior specificity for renal recurrence (73% vs. 18%; P = 0.006) (117, 118)s. Renal biopsy analyses confirm that IgG1 and IgG2 are the predominant subclasses deposited in glomeruli in LN patients, providing direct histopathological evidence of their pathogenic role in immune complex−mediated injury. Similarly, anti-C1q autoantibodies exhibit marked IgG subclass restriction: in lupus nephritis, over 30% are exclusively IgG2, and IgG2 dominance correlates significantly with elevated serum creatinine, profound C3 depletion, and more severe histopathological activity. Conversely, IgG3 anti-C1q seroreversion occurs uniformly during clinical remission (100% negativity), establishing IgG3 as a highly dynamic and activity−specific biomarker (119, 120). Regarding glycosylation, global IgG Fc hypogalactosylation is consistently observed in LN and mechanistically promotes complement activation, FcγR−mediated leukocyte infiltration, and subsequent parenchymal damage (120). Collectively, integrating IgG subclass profiling and site−specific glycosylation analysis into routine autoantibody assessment substantially enhances risk prediction accuracy and informs mechanism−targeted therapeutic strategies.

While each autoantibody parameter contributes uniquely to disease activity monitoring, severity grading, early identification of atypical phenotypes (e.g., APS−associated nephropathy), and discovery of novel pathogenic targets, no single biomarker adequately captures the immunopathological complexity of LN. Thus, the integration of multi−antibody panel models, high−throughput detection technologies, and multi−omics data represents a necessary pathway toward precise stratification and personalized management.

## 5 Multi−parameter integration and technological enabling: advancing toward precision stratification

### 5.1 Multi-antibody panel models: pathological classification and precision stratification

**Figure 2:**
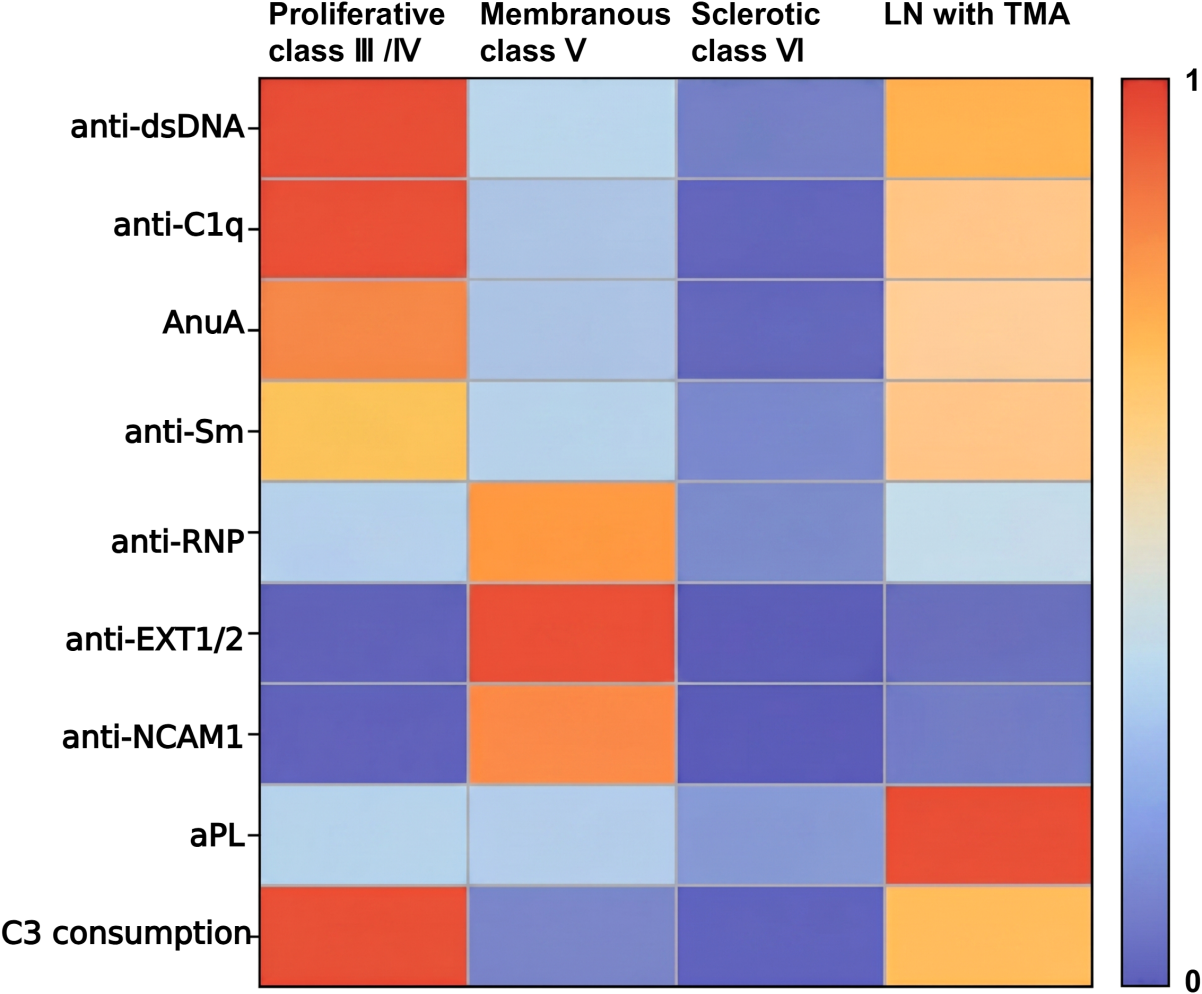
Multi-antibody panel heatmap for LN subtyping. Relative levels (0–1) of nine serological markers across proliferative (III/IV), membranous (V), sclerotic (VI), and TMA-associated LN. Proliferative LN: high anti-dsDNA, anti-C1q, AnuA, and C3 consumption. Membranous LN: elevated anti-U1-RNP, anti-EXT1/2, anti-NCAM1. Sclerotic LN: globally low levels. TMA-LN: high aPL with moderate complement consumption and anti-dsDNA/anti-C1q.

Serological antibody profiling offers valuable, non−invasive insights for the differential diagnosis and activity assessment of LN, particularly proliferative LN (classes III and IV) (**Figure 2**). Although anti−dsDNA antibodies remain a hallmark serological marker of SLE, their utility in distinguishing LN histological classes or reliably reflecting renal disease activity is limited by insufficient specificity (121). In contrast, the concurrent presence of high−titer anti−dsDNA antibodies, anti−C1q antibodies, and hypocomplementemia—termed the “serological triad”—shows strong clinical correlation with active proliferative LN. This triad aligns closely with defining histopathological features, including marked endocapillary hypercellularity, cellular crescent formation, and extensive subendothelial immune complex deposition. When timely renal biopsy is not feasible, detection of this triad serves as a pragmatic surrogate indicator of aggressive disease progression, enabling clinicians to initiate or escalate immunosuppressive therapy without delay (61). Furthermore, anti−nucleosome (AnuA) and anti−RNP70 antibodies show emerging discriminatory potential between proliferative and membranous LN (class V) (8). Notably, ANCA, especially p−ANCA, are not only associated with increased risk of LN development but also correlate significantly with higher SLEDAI scores, elevated anti−dsDNA titers, and more severe renal involvement (122).

Patients with membranous LN (class V) typically exhibit lower serological disease activity than those with proliferative LN, including reduced prevalence and titers of anti−dsDNA and anti−C1q antibodies, as well as milder complement depletion (32, 123). The pathogenesis of class V LN is predominantly driven by Th2−skewed humoral immunity, characterized by diffuse subepithelial immune complex deposition rather than prominent inflammatory cell infiltration. Importantly, some patients present with classic membranous nephropathy−like histology, including granular IgG and C3 deposits along the glomerular basement membrane, without hypocomplementemia or elevated anti−dsDNA levels, underscoring the need for heightened clinical suspicion of class V LN in such cases (123). Emerging evidence implicates NCAM1 as a novel autoantigen in LN; NCAM1 autoantibodies are detected in approximately 6.6% of LN patients, with titers potentially correlating with disease activity and showing responsiveness to rituximab therapy (124, 125). Moreover, aPL positivity in class V LN raises concern for concomitant antiphospholipid antibody−associated nephropathy, a condition linked to accelerated renal microvascular injury and poorer long−term renal outcomes (35). Complement component C4d deposition in renal arterioles has been validated as a specific biomarker of renal microvascular lesions (RVLs) and constitutes an independent predictor of adverse renal prognosis (126).

In advanced sclerotic LN (class VI), defined by global glomerulosclerosis involving >90% of glomeruli, the disease has transitioned to an irreversible chronic end−stage. At this stage, systemic immune activity is generally attenuated or exhausted; consequently, conventional serological markers, including anti−dsDNA antibodies, often decline in titer or become undetectable (127). As a result, the association between serological parameters (e.g., anti−dsDNA levels or complement C3/C4 concentrations) and histological or functional renal activity markedly weakens or dissociates entirely. Relying solely on these serological indices to guide therapeutic decisions in class VI LN is therefore clinically unreliable. Instead, progressive renal functional decline primarily reflects cumulative, non−inflammatory fibrotic damage. Prognostic evaluation should accordingly prioritize histopathological chronicity indices, such as the proportion of globally sclerotic glomeruli, severity of interstitial fibrosis, and degree of tubular atrophy (128). Recognizing this serology−pathology dissociation is critical to prevent unwarranted intensification of immunosuppression and its attendant risks (e.g., infection, malignancy). While anti−angiotensin II type 2 receptor (AT2R) antibodies are significantly elevated in LN patients relative to healthy controls and individuals with other glomerulopathies (129), their longitudinal association with renal disease course remains undefined. Novel targeted biologics, including telitacicept, a dual inhibitor of BAFF and APRIL, modulate B−cell−driven humoral immunity and represent promising therapeutic strategies for disease modification (130). Accordingly, management of class VI LN should emphasize supportive care, including rigorous control of hypertension and proteinuria, to mitigate further functional deterioration, rather than pursuing normalization of immunological serum markers.

Given these complexities, the development of multi−analyte serological models and integrated scoring systems has emerged as a pivotal focus in translational LN research. The principal clinical value of these multimodal models lies in enabling risk−adapted, precision−guided therapeutic decision−making. Integrated analysis improves identification of patients at highest risk for renal relapse (131). For example, a composite score incorporating high−titer anti−dsDNA, low C3, and p−ANCA positivity robustly predicts active proliferative glomerulonephritis and imminent recurrence (8, 117), thereby supporting escalation to B−cell−depleting agents (132) or pathway−specific interventions (e.g., TLR7 inhibition) in high−risk cohorts (133). Critically, serial score assessment allows real−time adjustment of treatment intensity, minimizing both undertreatment and overtreatment, and ultimately optimizes long−term renal preservation in LN.

### 5.2 Novel detection methods: high-throughput screening and molecular typing

Implementation of multi−analyte serological profiling necessitates advanced technological platforms, making high−plex autoantibody detection a cornerstone of translational research in LN. Automated immunoassay systems based on particle−based multiplex analysis technology (PMAT), for instance, allow simultaneous quantification of multiple autoantibodies, thereby substantially improving analytical throughput, reproducibility, and clinical scalability. Moreover, PMAT exhibits superior specificity for antinuclear antibody (ANA) detection compared with conventional indirect immunofluorescence (IIF), facilitating more robust stratification of patients into clinically and immunologically distinct SLE/LN subphenotypes (134). Similarly, high−throughput protein microarrays such as the i−Ome Discovery system have enabled unbiased discovery of novel IgG and IgA autoantibodies targeting DNA/RNA−binding proteins (e.g., LIN28A and HNRNPA2B1) in SLE cohorts, thereby expanding the repertoire of candidate biomarkers for precision diagnosis and disease monitoring (135). Luminex−based multiplex immunoassays have likewise proven instrumental in identifying disease−specific autoantibody signatures and optimizing diagnostic marker panels, notably in Sjögren’s syndrome, and hold parallel promise for LN biomarker validation (136).

Fine−specificity autoantibody profiling further elucidates context−dependent molecular pathogenesis. For example, α−enolase autoantibodies exhibit disease−specific isotype distributions and epitope recognition patterns: in LN, IgG reactivity targets conformational epitopes associated with neutrophil extracellular trap (NET) components, whereas in rheumatoid arthritis or vasculitis, distinct linear epitopes predominate (110). Complementary serum soluble mediator profiling reveals that elevated levels of vascular cell adhesion molecule−1 (VCAM−1) and syndecan−1 correlate significantly with both histological activity indices (e.g., activity index scores) and chronicity features (e.g., interstitial fibrosis) in LN kidney biopsies (137). Human cell−based microarrays represent a physiologically relevant platform for detecting conformationally intact and post−translationally modified (e.g., citrullinated, phosphorylated) autoantigens, thereby capturing autoantibody specificities inaccessible to recombinant protein arrays (138). Integrating high−throughput autoantibody profiling with unsupervised molecular clustering has enabled data−driven subclassification of LN patients into four immunologically and clinically discrete endotypes, each characterized by unique autoantibody repertoires, cytokine/chemokine profiles, and renal outcomes—outperforming conventional histopathological classification in predicting flare risk and differential treatment responsiveness (137). Ultimately, the convergence of multi−omics approaches, including deep proteomics, epigenomic profiling (e.g., DNA methylation landscapes) (139), and non−coding RNA analyses (e.g., circular RNAs and long non−coding RNAs) (140, 141), is poised to yield integrative, dynamic risk prediction models. Such models aim to identify patients at highest risk for rapid renal functional decline before irreversible structural damage occurs, thereby enabling preemptive, mechanism−informed therapeutic escalation or pathway−targeted intervention.

In recent years, advances in single-cell and spatial transcriptomics have enabled unprecedented resolution in characterizing the intrarenal immune microenvironment in lupus nephritis (LN). Single-cell RNA sequencing (scRNA-seq) of LN kidney biopsies has delineated functionally distinct immune cell subsets, including CD163+ dendritic cells (DC3s), CD4+ and CD8+ T lymphocytes, B cells, and heterogeneous myeloid populations, within the renal parenchyma. Notably, DC3s are markedly enriched in active LN kidneys and exhibit a strong positive correlation with clinical disease activity indices (e.g., SLEDAI-2K) and histopathological activity scores (e.g., ISN/RPS activity index). Mechanistically, DC3s engage in cognate interactions with CD4+ T cells via MHC class II-dependent antigen presentation, driving local clonal expansion and promoting Th1- and Th17-polarized differentiation—thereby amplifying intrarenal inflammatory cascades (142). Complementary spatial transcriptomics analyses have mapped the anatomical organization of these infiltrates, revealing structured immune-stromal crosstalk: for instance, peritubular fibroblasts and endothelial cells adjacent to T-cell–rich foci upregulate pro-inflammatory cytokines (e.g., CXCL9, IL-15) and fibrogenic mediators (e.g., TGFB1, PDGFA), forming functional niches that sustain inflammation and initiate tubulointerstitial fibrosis (143).

Integrating spatial transcriptomics with high-resolution proteomics enables precise localization of the anatomical microenvironment of autoantibody-producing cells in the tubulointerstitial and periglomerular regions. For instance, spatial transcriptomics can identify the specific spatial distribution within the kidney of follicular helper T cells (Tfh) expressing CXCR5, BCL6, and PD-1, as well as CD138^+^plasma cells and memory B cells (144). Meanwhile, combining laser capture microdissection (LCM) with liquid chromatography-tandem mass spectrometry (LC-MS/MS) allows direct extraction of proteomic information from defined microregions of the same tissue section, enabling identification of locally produced autoantibody profiles (e.g., anti-dsDNA, anti-C1q, anti-nucleosome antibodies) and activated complement fragments (C3a, C5b-9) (145). This integrated approach has revealed in active lupus nephritis (LN) patients that Tfh and plasma cells form functional “lymphoid aggregates” within the renal interstitium, where deposited immunoglobulins around these structures show significant correlation with corresponding serum autoantibody titers—such associations are rarely observed in non-inflammatory areas (146). These findings provide direct histological evidence for how circulating autoantibodies cross the endothelial barrier, localize to specific renal compartments, and drive local tissue damage. By integrating single-cell immune subset characteristics, spatial localization data, proteomic profiles, and circulating autoantibody signatures, it is now possible to construct a three-dimensional dynamic model linking systemic immune activation to focal renal injury, paving the way for novel strategies in early diagnosis, targeted therapeutic decision-making, and precision interventions based on the intrarenal immune microenvironment. Integrating single-cell immune subset characteristics with autoantibody profiling data holds promise for more precise decision-making in the early diagnosis and targeted treatment of LN.

## 6 Current challenges, clinical decision support, and future directions

### 6.1 Major challenges in clinical translation

The clinical translation of autoantibody profiling in lupus nephritis (LN) confronts three interrelated challenges. First, analytical standardization remains a critical barrier. Detection of established serological markers, including anti-dsDNA antibodies, is performed using heterogeneous methodologies (e.g., ELISA, chemiluminescence immunoassay (CLIA), and the Farr assay), each differing in antigen presentation, binding kinetics, and analytical sensitivity. These methodological disparities contribute to inter-assay variability, impeding the establishment of universally applicable clinical decision thresholds and compromising longitudinal data comparability. Moreover, non-specific binding inherent to certain platforms diminishes the robustness of observed associations between antibody levels and histopathological features of renal injury (7). Second, substantial inter-individual heterogeneity and temporal dynamism characterize LN autoantibody repertoires. Antibody profiles exhibit marked patient-to-patient variation and evolve dynamically across disease phases. Although epidemiological associations exist, such as elevated anti-nucleosome antibodies in proliferative LN and anti-RNP70 antibodies in membranous-pattern, these correlations lack absolute specificity, with considerable overlap in seropositivity rates across histological classes (8). Similarly, emerging autoantibodies, including anti-C3, anti-C1q, and anti-mCRP, display context-dependent expression patterns; their clinical interpretation necessitates integration with comprehensive clinical context, histopathological subclassification, and therapeutic timeline (10, 147, 148). Third, a paucity of high-quality prospective evidence limits clinical utility. The majority of current evidence derives from cross-sectional or retrospective cohort studies, which establish associative, not causal, relationships between specific autoantibody profiles and clinical phenotypes (e.g., proliferative histology) or treatment outcomes (149). Crucially, large-scale, randomized interventional trials validating whether autoantibody-guided decisions improve hard clinical endpoints, such as progression to ESRD or all-cause mortality, remain absent (56). Consequently, autoantibody profiling currently serves primarily as an adjunctive diagnostic tool and has yet to be embedded within validated, mechanism-informed clinical decision pathways for LN management.

### 6.2 Construction of a clinical decision-making framework based on antibody profiles and the development of precision medicine

**Figure 3:**
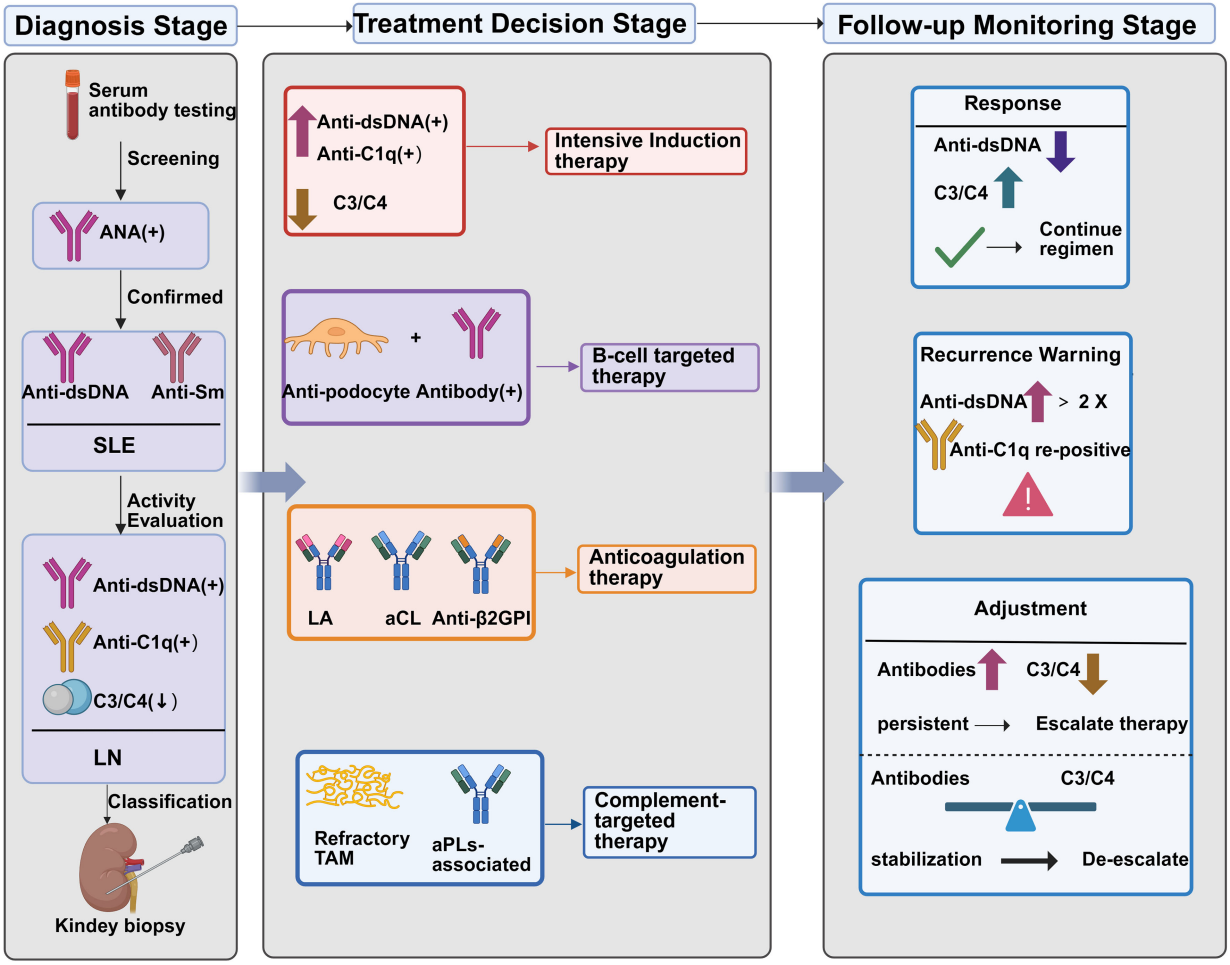
Autoantibody profile-based framework for comprehensive management of lupus nephritis. This framework integrates autoantibody profiling throughout the three core phases of LN management, i.e., diagnosis, therapeutic decision-making, and longitudinal follow-up, guiding risk stratification, individualized therapy, and dynamic prognostic assessment.

In the comprehensive, longitudinal management of LN, a clinical decision framework anchored in autoantibody profiling is driving a paradigm shift from empiric, population−based treatment toward mechanism−informed, individualized strategies (**Figure 3**). This framework operates across three clinically defined phases: diagnosis, therapeutic decision−making, and longitudinal monitoring.

During diagnosis, although ANAs remain highly sensitive screening tools for SLE, their limited specificity necessitates confirmatory testing with high−specificity autoantibodies, including anti−dsDNA and anti−Sm antibodies, to establish a definitive SLE diagnosis and support LN (150). Anti−C1q antibodies have emerged as robust activity−associated biomarkers in LN, showing strong correlation with renal flare and histological activity (73). Integrating multi−autoantibody profiling into hierarchical diagnostic algorithms, and leveraging natural language processing (NLP) to systematically extract structured serological data from unstructured electronic health records (EHRs), enhances spectral completeness and analytical rigor, thereby strengthening the foundation for precision phenotyping and early intervention (151).

In therapeutic decision−making, specific serological signatures serve as actionable immunological indicators. Persistent high−titer anti−dsDNA positivity, coupled with anti−C1q seropositivity and hypocomplementemia, reflects sustained classical complement pathway activation and immune complex deposition, supporting timely initiation of intensified induction regimens and identifying candidates most likely to benefit from prompt, targeted immunosuppression (73, 150). Furthermore, autoantibody profiles, including patterns of B−cell epitope reactivity and isotype distribution, inform the rational selection of B−cell−targeted biologics (e.g., rituximab, belimumab, and emerging anti−BAFF/APRIL agents), aligning therapy with underlying humoral pathobiology (152, 153). For LN patients positive for aPLs, especially those with lupus anticoagulant (LA) positivity, triple positivity, or anti−β2GPI−D1 positivity, a high index of suspicion for coexisting renal TMA is warranted. Such patients should receive anticoagulation in addition to immunosuppressive therapy; in refractory cases, the anti−C5 monoclonal antibody eculizumab may be considered (94–99).

During longitudinal monitoring, serial quantification of anti−dsDNA and anti−C1q antibody titers, along with dynamic assessment of complement C3 and C4 levels, provides objective, real−time metrics for evaluating treatment efficacy, forecasting impending renal flares, and guiding adaptive adjustments to maintenance immunosuppression (73, 147, 150). This enables a transition from rigid, time−fixed treatment protocols to responsive, biomarker−guided management, in which therapeutic intensity is dynamically calibrated to individual immunological trajectories, thereby optimizing the risk−benefit balance and preserving long−term renal function.

### 6.3 Prospective targeted therapy trials

Prospective clinical trials that stratify patients by autoantibody profiles are essential to rigorously validate the clinical utility and therapeutic superiority of precision medicine approaches in LN, as current standard-of-care management remains largely empirical, relying on broad-spectrum immunosuppression that yields suboptimal complete response rates and carries substantial risks of infection, metabolic toxicity, and malignancy (154). Enriching trial populations with validated autoantibody biomarkers improves statistical power, accelerates recruitment, and enhances the biological plausibility and clinical interpretability of treatment effects. For example, elevated baseline anti−C1q antibody titers robustly predict complete renal response to conventional induction therapy (150), supporting the rational design of phase II trials evaluating complement classical pathway inhibitors or selective B−cell activation modulators specifically in anti−C1q−positive LN patients. Similarly, integrative autoantibody clustering has identified a high−risk pediatric LN endotype characterized by concurrent anti−dsDNA and anti−Ro/SSA positivity, an endotype associated with a markedly increased risk of ESRD (9, 56, 147), and prospective interventional studies testing intensified, mechanism−tailored immunosuppression (e.g., early rituximab escalation or calcineurin inhibitor optimization) in this molecularly defined cohort hold strong potential to improve long−term renal survival. Furthermore, promising therapeutic targets emerging from mechanistic research, including TLR7 antagonists (134) and cGAS−STING pathway inhibitors (155), require formal validation through adequately powered, randomized phase II/III trials. Critically, serial quantification of autoantibody dynamics (e.g., anti−dsDNA decline kinetics, anti−C1q normalization) should be prospectively incorporated as pharmacodynamic biomarkers to assess target engagement and biological activity, thereby evolving autoantibody profiling from a static diagnostic and prognostic tool into a dynamic, decision−supportive biomarker that guides real−time therapeutic adaptation.

### 6.4 Multi−omics integration and construction of artificial intelligence−based predictive models

**Figure 4:**
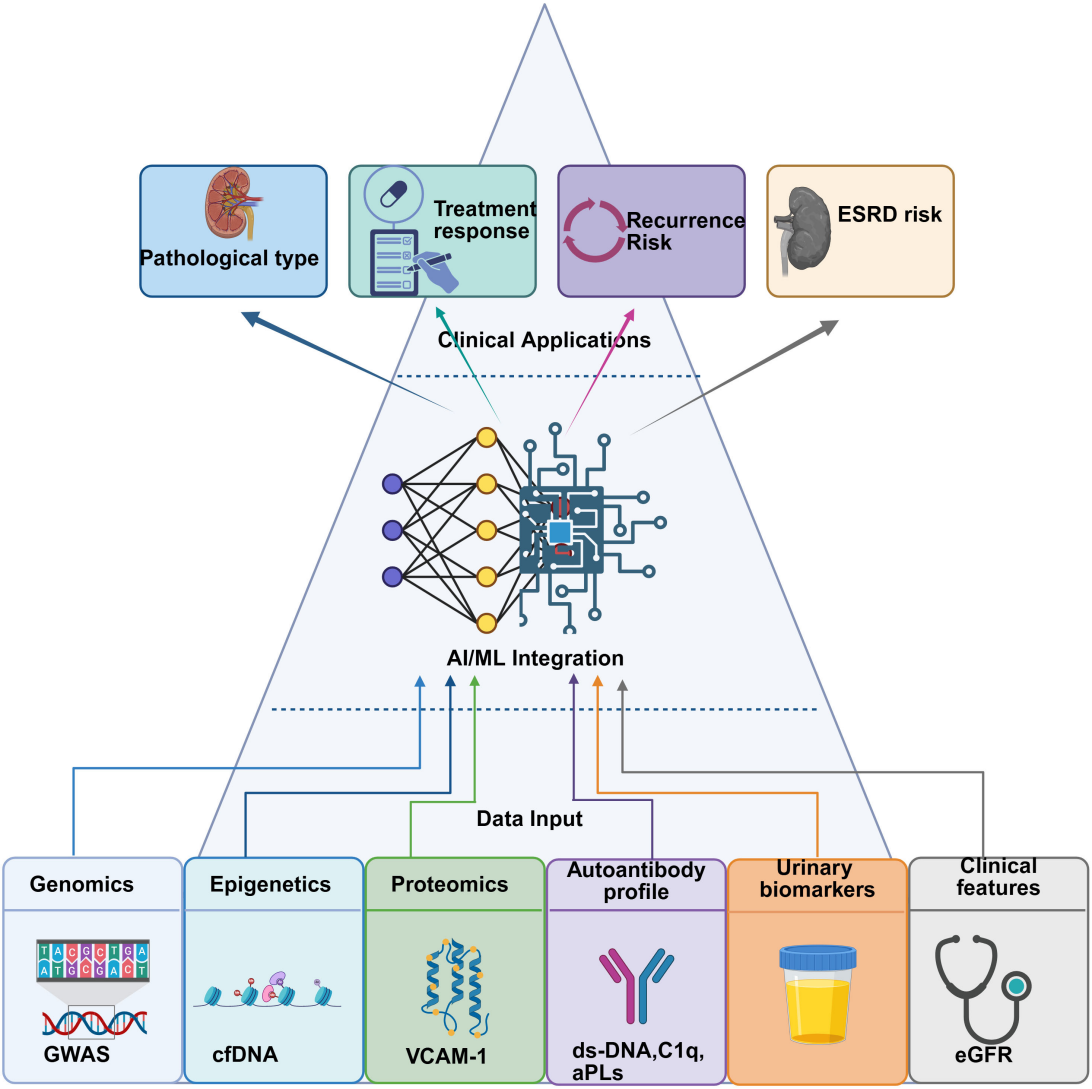
Multi−omics integration and artificial intelligence−based predictive model framework. A hierarchical pyramid structure illustrating the core pathway for future risk prediction and precision management of lupus nephritis (LN). By integrating multidimensional biological data (genomics, epigenomics, proteomics, transcriptomics, etc.) with clinical information, and leveraging artificial intelligence and machine learning algorithms, a comprehensive predictive model is constructed. This framework drives the transition of LN management from reactive treatment toward proactive, predictive, and individualized precision medicine.

Integrating autoantibody profiling with multi-omics data to construct comprehensive artificial intelligence-based predictive models represents a frontier direction for future risk prediction and management in LN (**Figure 4**). At the genomic level, genome-wide association studies (GWAS) have robustly identified susceptibility and severity-associated loci (e.g., HLA-DR2/DR3, IRF5, STAT4), with polygenic risk scores increasingly informing early identification of high-risk SLE patients (154, 156, 157). Epigenomic analyses reveal disease-specific hypomethylation patterns in cell-free DNA from plasma, which correlate strongly with global disease activity and renal flare risk, suggesting utility as a minimally invasive longitudinal biomarker (158). Urinary proteomics provides direct insight into intrarenal inflammation: markers reflecting monocyte/neutrophil degranulation and macrophage activation exhibit high concordance with histopathological classification (e.g., proliferative vs. membranous) and activity indices, and their early modulation predicts subsequent treatment response (159). Complementary single-cell and bulk RNA sequencing of urinary immune cells further identifies predictive cellular signatures, including activated effector memory CD4+ T cells and macrophage-derived transcriptional modules, that precede clinical improvement by weeks (160). Serum soluble mediators, including syndecan-1 and VCAM-1, demonstrate differential expression across LN histological subtypes and independently predict induction therapy outcomes (161). Machine learning and deep learning frameworks are now being leveraged to fuse these heterogeneous, high-dimensional datasets into unified predictive algorithms. These models generate individualized, multimodal risk scores that prospectively classify renal histopathology, quantify real-time disease activity, forecast treatment responsiveness, and estimate long-term renal prognosis—thereby enabling proactive, mechanism-informed patient stratification and adaptive therapeutic escalation or de-escalation. This integrative paradigm shifts LN management from reactive, histology-dependent care toward predictive, biomarker-guided precision medicine.

Artificial intelligence and machine learning algorithms provide powerful tools for integrating these massive multi-omics datasets, enabling the construction of comprehensive scoring systems that predict individual patient renal pathology class, disease activity, treatment response, and long-term prognosis. This facilitates precise patient stratification and dynamic risk monitoring (154, 161), ultimately driving the transformation of LN clinical practice from passive, one-size-fits-all therapy toward proactive, predictive, and individualized precision management.

## 7 Conclusion

The role of the autoantibody repertoire in LN has evolved from a conventional diagnostic tool into a dynamic, multidimensional biomarker system, marking a paradigm shift from “static label” to “dynamic participant”: autoantibodies are not merely markers of disease classification but also key effector molecules driving renal injury and real−time windows reflecting disease activity. At the clinical level, classic markers such as anti−dsDNA and anti−C1q antibodies have established a central role in assessing the activity of proliferative lesions, with their dynamic titer changes closely linked to the risk of disease relapse. The antiphospholipid antibody profile provides irreplaceable early warning for critical pathological phenotypes such as thrombotic microangiopathy. Clinical interpretation must move beyond binary positive/negative judgments and integrate autoantibody findings within specific clinical and pathological contexts. From a mechanistic integration perspective, distinct autoantibody combinations may point to different immunopathogenic pathways and histopathological classes. Future directions lie in constructing multidimensional evaluation models that integrate multiple autoantibodies, complement levels, specific protein markers, and clinical features, thereby enabling non−invasive “liquid biopsy” that accurately reflects the real−time intrarenal immune status and provides evidence−based support for stratified therapy. Future efforts should also focus on refined antibody characteristics, including IgG subclass and glycosylation modifications, incorporate insights into the renal immune microenvironment revealed by single−cell and spatial transcriptomics, and leverage artificial intelligence to integrate multi−omics data, ultimately achieving more precise stratification and individualized treatment of LN.

However, clinical translation still faces significant challenges. Standardization of detection methods is the cornerstone of reliable comparison and result interpretation; inter−laboratory variability may affect the accuracy and comparability of markers. The association between dynamic autoantibody changes and individual prognosis requires high−level evidence from large−scale prospective cohort studies to clarify which autoantibody profile alterations can reliably guide treatment escalation or de−escalation. Bridging the gap from correlation to causation represents a core bottleneck in advancing autoantibody profiling from an auxiliary diagnostic tool to a basis for therapeutic decision−making. Looking forward, multidisciplinary collaboration among nephrology, rheumatology, laboratory medicine, and bioinformatics is urgently needed. Through standardized detection methods, deeper mechanistic studies, and prospective interventional trials, autoantibody−based individualized precision management can move from theoretical frameworks to routine clinical practice, ultimately improving long−term renal survival and quality of life in LN patients and achieving the transition from empirical therapy to precision medicine.
